# Supplementary material for: Are carbon emissions trading and green financial instruments synergistic? -Comprehensive quantitative research based on content analysis
Source: PLoS One. 2024 Mar 7;19(3):e0298601. doi: 10.1371/journal.pone.0298601 (PMC10919601; doi:10.1371/journal.pone.0298601)
Supplement: S1 Data — (ZIP) [file pone.0298601.s001.zip › Data/地级市Z_F工作报告.zip!/║╙─╧/╔╠╟≡╩╨/└┤╫╘╣1⁄2╓┌║┼í╛┬φ┐╦╩2╛▌═°í┐_2017.docx]

各位代表：

　　现在，我代表市人民政府，向大会报告工作，请予审议，并请各位政协委员和其他列席人员提出意见。

　　 一、过去五年工作回顾

　　市四届人大一次会议以来，面对复杂多变的经济形势和繁重的发展任务，在省委、省政府和市委的正确领导下，全市上下牢固树立和落实新发展理念，坚持“持续求进、好中求快”的工作总基调，着力稳增长、调结构、促改革、扩开放、控风险、惠民生，经济社会保持了又好又快发展的良好态势，圆满完成了市四届人大一次会议确定的各项目标任务。

　　这五年，经济社会加快发展，综合实力不断增强。以提高经济发展质量和效益为目标，加快转变发展方式，经济保持平稳较快发展。2016年，全市生产总值达到1974亿元，增速跃居全省第3位，年均增长9．6％；一般公共预算收入117亿元，总量较2011年翻了一番；规模以上工业增加值775亿元，增速居全省第3位；社会消费品零售总额919亿元，较2011年翻了一番，增速跃居全省第1位；固定资产投资1991亿元，较2011年翻了一番；城镇居民人均可支配收入和农民人均纯收入年均分别增长9．8％和11％，高于经济增长速度；节能减排完成省定目标任务。三次产业结构调整为19．6∶41．2∶39．2，一产占比首次降到20％以下，三产提高11个百分点。2016年全市18项主要经济指标，有16项增速居全省前5位，实现了“十三五”良好开局。

　　这五年，转型升级步伐加快，现代产业体系不断完善。坚持优化存量、做强增量，产业结构调整取得积极成效，现代产业体系不断完善。工业转型升级取得新突破。五年新增规模以上工业企业603家，实现主营业务收入3520亿元，年均增长13．3％。产业集聚区建成区面积较2011年增加35．4平方公里，入驻各类企业2487家。纺织服装制鞋、食品、装备制造3个千亿级产业集群，主营业务收入分别达到900亿元、821亿元和813亿元。初步建成了中国制冷产业基地、制鞋产业基地、超硬材料基地等9个百亿级特色产业集群，基本形成布局合理、特色鲜明的发展格局。4个产业集聚区晋升二星级，5个晋升一星级；1个服务业“两区”晋升三星级，5个晋升一星级。在全省产业集聚区观摩中，我市连续7年获得小组第一名。农业产业化加快推进。五年累计建成高标准粮田411．3万亩，粮食产量常年稳定在120亿斤以上，连续三年荣获“全国粮食生产先进单位”。打造了夏邑食用菌、柘城三樱椒、宁陵酥梨、民权河蟹、梁园草莓等一批知名农产品品牌，畜牧养殖业在全省领先地位进一步巩固。农业机械化水平较2011年提高10个百分点。发展专业合作社、家庭农场等新型经营主体9200多家，新培育市级以上农业产业化龙头企业25家、省级农业产业化集群12个，五得利、高老庄、贵友等一批农产品品牌企业健康发展。现代服务业快速发展。五年累计完成服务业增加值2894亿元，年均增长10．8％。新入驻规模以上服务业企业595家，传化物流等一批物流项目开工建设，快递业务量年均增长92．6％，各类市场销售总额突破700亿元，建成电子商务产业园7个。阿里巴巴·商丘产业带入驻企业1300余家，累计交易额20亿元。成功引进32家金融机构，科迪乳业在主板上市，5家企业在“新三板”挂牌。全域旅游加快发展，启动商丘古城修复性保护与展示工程，东南城墙、陈家大院、工业遗产园等项目修复完成，芒砀山旅游区成功创建5A级景区、睢县北湖创建4A级景区，新增国家级水利风景区6处，民权县荣获国家级全域旅游示范区。

　　这五年，发展基础持续巩固，城乡面貌不断改观。坚持抓重点、补短板，五年累计完成固定资产投资7473亿元，年均增长19．2％。交通优势更加彰显。郑徐高铁建成通车，商合杭高铁开工建设；商周高速二期、郑民、商登高速建成通车，连霍高速商丘段改扩建工程加速推进，国道310和105完成改造，五年新增高速公路134公里，新建、改扩建国省道401公里、农村公路3948公里，高等级公路网密度位居全省第一。城市基础设施不断完善。新建供水管网90多公里，公共供水覆盖面逐年扩大。累计铺设供热管网230多公里，实际供热面积达390万平方米，2×35万千瓦热电联产项目进展顺利。中心城区新建污水处理厂3座，污水处理能力达到33万吨。能源保障水平进一步提升。新建各类输变电站109座，架设供电线路671．5公里，供电能力进一步增强。建成西气东输豫东支线，累计铺设天然气管道1000多公里。水利基础设施不断完善。完成了三义寨引黄灌区续建配套与节水改造工程、黄河故道水库除险加固、中小河流治理等水利工程项目建设，水资源保障能力不断提升。信息支撑能力显著增强。建成首个云计算大数据中心和全国百万级“全光网城市”，被评为2016年度“宽带中国”示范城市。新型城镇化进程不断加快。全市城镇化率达到40％，五年提高8．5个百分点。中心城区功能不断完善，五年完成基础设施投资349亿元，新建、改造道路200余条，打通断头路32条，归德路、凯旋路跨陇海铁路和郑庄立交建成通车，商丘高铁站等一批标志性工程竣工投用；疏浚内河河道150多公里，黑臭水体得到初步治理；完成了两个火车站广场升级改造，日月湖景区、汉梁文化公园一期、华夏游乐园等大型公园建成开放，建成一批廊道绿化和街头绿地，五年新增城市绿地547万平方米；加强市容环境设施建设，新建公厕220座、垃圾中转站47座、环卫工人服务站19座；购置各类环卫设备1200多台，主次干道机械化清扫率达到100％；新增公交线路58条，投放新能源公交车1000辆、公共自行车3000辆。大棚户区改造步伐加快，2016年争取国家棚改计划11．9万套，占全省任务的28％，到位国家财政补助资金9．6亿元。深入开展“六城”联创，勇夺全国双拥模范城七连冠，成功创建国家森林城市；顺利通过省级卫生城复核，成功创建省级文明城；永城、睢县、夏邑成功创建国家园林城。中心城区常住人口超过125万人，永城达到55万人，夏邑、虞城超过30万人；建制镇发展到84个，15个镇跻身全国重点镇，6个镇被列为省级经济发达镇行政管理体制改革试点。以农村环境综合整治为突破口，扎实开展“美丽商丘·整洁村镇”活动，598个村达到省定改善人居环境示范村标准。

　　这五年，改革开放深入推进，发展活力不断释放。坚持以改革破解难题。全面完成政府机构改革，稳步推进事业单位分类改革，深入推进“放管服”及商事制度改革，取消、下放行政审批事项180项，市级保留100项，是全省保留行政审批项目最少的省辖市之一；全市市场主体发展到28．5万户，居全省第4位；推进智慧政务网络建设，政务服务水平不断提升；整合公共资源交易平台，组建市级公共资源交易中心。深化投融资体制改革，五年融资326．8亿元，资金保障能力不断增强。初步理顺了城乡一体化示范区管理体制；完成了产业集聚区与乡镇行政区域管理套合。完成876万亩农村土地承包经营权确权登记颁证；全面放开城镇落户限制，五年转移农村人口80．8万人。国有企业、医药卫生、农村综合改革等取得积极进展。坚持以创新激发活力。创建国家级高新技术产业化基地3家、国家级科技企业孵化器4家、国家级检测中心3家、“院士工作站”2个；发展高新技术企业54家、省级工程技术研究中心45家；省级高新区达到4家，居全省第1位。积极实施开放招商。成功举办3届国际华商节，累计利用省外资金2441亿元，落地3亿元以上项目185个、总投资1635亿元，安踏、赛琪、万宝等一批知名企业落户商丘。开放平台建设取得重大突破，保税物流中心通过海关总署联合验收，国家一类铁路口岸和综合保税区纳入全省专项规划。

　　这五年，社会事业全面发展，人民生活水平不断提高。坚持办好省重点民生工程和市定十件实事，五年投入各类民生资金1397．6亿元，占财政支出比重达到79．6％。脱贫攻坚取得阶段性成果。把脱贫攻坚作为重要政治任务，累计投入扶贫资金51．5亿元，整合各类资源，精准发力，精准施策，以产业扶贫为重点，统筹推进教育、健康等扶持措施，603个村实施整村推进，67．8万人实现脱贫。大气污染防治成效显著。取缔小散乱污企业1082家，清理砖瓦窑厂198家，关停“黑加油站点”898座，完成5300家餐饮企业油烟净化装置改造，倒逼企业加大环保设施投入，改进生产工艺。通过实施9项精准治理措施，三项指标在全省位次不断前移，“商丘蓝”刷爆“朋友圈”，我市大气污染防治工作得到省委、省政府充分肯定。社会保障水平不断提升。连续四年五次提高城乡低保救助标准，累计发放低保资金31．2亿元；完成城乡居民养老保险制度并轨，医疗保险基本实现全覆盖；累计新增城镇就业40万人，农村劳动力转移就业35万多人。建成保障性住房7．1万套、354万平方米；发放住房公积金贷款44．7亿元，购房补贴3亿元。教育供给能力持续增强。新建改扩建中小学2071所、幼儿园904所，商丘幼师、商丘工学院新校区等建成投入使用，商丘医专、一高、一中、实验幼儿园新校区等一批学校开工建设。医疗卫生事业稳步推进。新建市第一人民医院儿童病房楼、市中医院综合病房楼、虞城县人民医院、睢县中医院等医疗卫生项目1294个，市第一人民医院成功创建三级甲等医院。文化服务体系不断完善。建成156个乡镇综合文化站、3899个农家书屋，放映农村公益电影25万余场，市博物馆建成并免费向公众开放，市文化艺术中心、体育中心开工建设，《龙河钟声》、《王昭君》分获省第十二届、十三届戏剧大赛文华奖；大运河商丘段申遗成功。社会治理方式不断创新。加强社会治安综合治理，做好矛盾预防化解，连续六年荣获全省“信访工作先进省辖市”，人民群众安全感和满意度不断提升。食品药品监管不断加强，没有发生重大食品药品安全事故。深化重点领域排查整顿，安全生产形势总体平稳。

　　这五年，民主法治扎实推进，精神文明建设不断加强。深入开展党的群众路线教育实践活动、“三严三实”“两学一做”专题教育等，政府自身建设得到加强。自觉接受人大法律监督、政协民主监督和社会各界监督，办理人大代表建议和政协委员提案3858件。深入推进依法行政，运用法治思维、法治方式处理问题的能力得到增强。深入推进机关作风建设，全面建立权责清单和负面清单，政府服务发展、服务群众的能力进一步增强。圆满完成“六五”普法任务，完成第八届村委会和第四届、第五届居委会换届选举工作。加强社会主义核心价值观教育，开展移风易俗、文明乡风活动，持续擦亮“商丘好人”品牌，新涌现“冰凌哥”“光膀哥”等一批正能量“网红”，22人荣登中国好人榜，7家单位被评为全国文明单位。持续加强国防教育、国防后备力量及人民防空建设，驻商解放军、武警部队和预备役部队为地方发展作出新贡献，双拥工作持续深化，军民融合深度发展。支持工会、共青团、妇联等人民团体开展工作。对口援疆和区域经济技术协作取得较好成效。气象、农林科研、残疾人事业、民族宗教、外事侨务、对台、史志、盐业、烟草、档案、防震减灾等工作取得新成绩。

　　各位代表！过去的五年是我们攻坚克难、砥砺奋进、加快发展的五年，是全市经济快速增长、城乡面貌显著变化、人民群众获得感不断增强的五年，是各项事业蓬勃发展、社会大局和谐稳定的五年。这些成绩的取得，是省委、省政府和市委正确领导的结果，是市人大、市政协监督支持的结果，更是广大干部职工和900多万商丘人民真抓实干、共同奋斗的结果。在此，我代表市人民政府，向全市人民，向全体人大代表、政协委员，向各民主党派、人民团体，向驻商部队、武警官兵、公安民警，向所有支持商丘建设发展的各界人士，致以崇高的敬意和衷心的感谢！

　　成绩来之不易，经验弥足珍贵。五年来，团结奋斗和实干兴商的生动实践，使我们深刻认识到：必须坚持发展第一要务。发展是解决一切问题的总钥匙。落实好发展这个“硬任务”，必须以提高质量和效益为中心，把创新、协调、绿色、开放、共享五大发展理念贯穿发展全过程和各领域，调动一切积极力量，凝聚推动发展的强大合力，走出一条传统农区转型发展之路。必须抓住项目这一载体。持续发挥好承接产业转移示范市的平台作用，始终坚持以项目建设推动发展，不断激活社会和民间投资，更加注重项目引进，促进产业基础更强、优势更优，走出一条项目带动、投资拉动的跨越发展之路。必须坚持改革这一方向。以供给侧结构性改革为统领，落实好“三去一降一补”五大任务，推动产业转型发展，实施战略性新兴产业倍增计划，努力实现发展速度、质量和效益相统一。全面破除阻碍发展的体制机制，为企业松绑，为创新除障，为公平护航，走出一条释放潜力、激发活力、增强动力的改革发展之路。必须牢记民生这一根本。人民对美好生活的向往，就是我们的奋斗目标。必须把改善民生作为一切工作的出发点和落脚点，坚持做好民生实事，不断提高基本公共服务水平，补足发展短板，甩掉贫困帽子，筑牢社会保障安全网，走出一条政策利民、发展惠民的民生发展之路。必须守住生态这一红线。当前，我市正处于全面建成小康社会的关键时期，经济下行压力加大，资源约束趋紧。正因如此，我们更应该守住生态保护这条红线，不断扩大环境污染防治攻坚成果，保护好生态环境，走出一条经济社会与生态文明互促互进的绿色发展之路。必须坚持务实这一要求。树立务实重干作风，认真落实深入基层、深入群众的工作要求，勇于担当、雷厉风行，遇到问题不回避、遇到困难不退缩，对定好的事情紧抓不放，对确定的目标逐项落实，对部署的任务一抓到底，走出一条“马上办抓落实”的务实发展之路。

　　看到成绩的同时，我们还清醒地认识到，我市经济社会发展还存在一些困难和问题。主要表现在：一是总量小、基础差、底子薄、发展不平衡的现状尚未根本改变，总体实力不强、发展不足、脱贫攻坚任务重仍是商丘面临的主要问题。二是产业结构不优、竞争力不强，高端人才匮乏，科技创新能力不足，资源环境约束加剧，转型攻坚任务繁重。三是基础设施不完善，辐射集聚能力弱，公共服务供给能力不足，距人民群众企盼还有较大差距。四是政务环境有待优化，办事效率有待提高，敢担当、能担当、善担当的能力有待提升。对此，我们将高度重视，在今后的工作中，采取有力措施，认真加以解决。

　 　二、今后五年政府工作的总体思路和奋斗目标

　　今后五年，是我市全面完成“十三五”规划的五年，是决胜全面小康、实现第一个百年奋斗目标的五年，更是我市抢抓机遇、爬坡过坎、夯实基础、乘势而上，实现又好又快跨越发展的五年。未来五年，国家将持续深化供给侧结构性改革，“一带一路”效应也将不断放大，新一轮国家城镇体系规划、综合交通规划、中部崛起规划等重大战略规划和重大战略布局，都明确了商丘在全国作为区域中心城市、综合交通枢纽城市、物流节点城市的定位和作用。我省在未来五年将整合资源，集中力量，加快推进“三区一群”等引领性、整体性、专题性战略，这些都为我们利用政策复制和经验推广，积极融入区域协同和创新发展战略提供了难得机遇。经过五年的快速发展，商丘已经处在了全面建成小康社会的历史新方位，站在了提质增效、转型发展的新起点，进入了加快发展、实现跨越的新阶段。市五次党代会和“十三五”规划为商丘发展描绘了宏伟蓝图，九项跨越发展行动计划明确了未来发展任务，全市上下思发展、谋发展、促发展，想干事、敢干事、能干事的氛围更加浓厚。只要我们保持战略定力、增强发展自信，发挥自身优势、抓住特有机遇，积极作为、奋力拼博，就一定能够掌握发展主动权，开辟发展新局面，实现发展新目标。

　　今后五年的指导思想是：以党的十八大、十八届历次全会和即将召开的十九大精神为指导，深入贯彻习近平总书记系列重要讲话和调研指导河南工作时的重要讲话精神，深入贯彻党中央治国理政新理念新思想新战略，紧紧围绕“五位一体”总体布局和“四个全面”战略布局，着力发挥优势打好“四张牌”，自觉践行五大发展理念，积极对接“三区一群”战略，按照市委“四个总”工作要求和“一六六一”发展思路，以提高发展质量和效益为中心，以推进供给侧结构性改革为主线，以项目建设为抓手，以枢纽经济为突破，着力建设经济强、城乡美、活力足、社会安、生态好、百姓富的幸福商丘，全面建成小康社会，顺利实现中国梦第一个百年奋斗目标。

　　今后五年的奋斗目标是：奋力实现“四个翻番”，全市产业集聚区主营业务收入和税收收入、第三产业增加值和税收收入实现翻番；“两个突破”，经济总量突破3000亿元，一般公共预算收入突破200亿元；“三个高于”，生产总值、一般公共预算收入、城乡居民收入增速高于全省和淮海经济区平均水平；“三个同步”，城乡居民收入与经济增长同步，生态环境质量改善与经济效益同步，社会事业与经济发展同步。

　　今后五年的发展愿景是：围绕上述奋斗目标，通过五年的共同努力，全力打造“六区”，实现“六个基本建成”，形成商丘又好又快跨越发展的强力支撑。

　　－－基本建成具有较强实力的国家区域中心城市。积极培育战略性新兴产业，以农业产业化为引领的农业基础地位更加巩固，以装备制造业为主导的现代工业体系更加完善，以商贸物流、总部经济、金融服务、创新创意、会展旅游等为主体的现代服务业体系基本建成，以枢纽经济为显著特征、符合商丘实际的产业结构更加合理、优势更加明显，综合竞争力不断提升。中心城区的首位度显著提升，重点功能区协同发展，对外开放度全面提升，要素资源更加集聚，辐射带动作用不断增强，基本建成区域健康医疗中心、科技教育创新中心、物流配送分拨中心、承接产业转移制造中心。

　　－－基本建成通达便捷的立体化综合交通枢纽。“十字”加放射贯通南北、连接东西、通达全国的高铁线网基本形成，网格状高速公路网络更加完善，“三环九放射”“六个城市出入口”“井字加环线”对接周边、全域畅通、高效便捷的快速路系统基本建成，适时推进中心城和市域轨道交通、城际铁路建设。构建多式联运、空铁路河一体的现代化立体交通体系，“一网四港六中心”枢纽布局基本形成。综合交通枢纽支撑和带动经济发展的能力显著增强，全国综合交通枢纽地位不断巩固。

　　－－基本建成城乡一体化发展的新格局。按照向心发展、协同发展、错位发展和重大基础设施统一规划建设的原则，加快形成“一中心两组团五卫星城百镇区”带动千村发展的以城带乡、城乡互补、融合互动的一体化发展格局。全市城镇化率年均增长1．8％左右，中心城区和县城实现市政基础设施“四率”全覆盖，永城区域副中心城市基本建成，柘城、民权、睢县县城人口规模达到30万人以上。强力推动城乡一体化示范区建设，发挥好在产业发展、城市建设等方面的引领作用，成为“生产空间集约高效、生活空间宜居适度、生态空间山清水秀”的典范。

　　－－基本建成环境优美的生态体系。环境污染防治攻坚成果得到巩固，市域河流水质全部达标，空气质量优良天数高于省定目标任务。巩固国家森林城市创建成果，森林覆盖率达到36％以上。五年新增公共绿地面积1500万平方米以上，城市人均公园绿地面积达到12平方米以上。争取早日启动引江济淮等重大水源建设工程，新增引水3．6亿立方米，水源保障能力显著提高。中心城区“九河三湖”水系及沿岸带状公园、大型文化公园、湿地公园、城市游园和街头绿地共同构成宜居生态城的重要支撑。基本形成以黄河故道为主屏障，特色浓郁、环境优美、宜居宜业“天蓝、水清、地绿”的全域生态体系。

　　－－基本建成富有活力的创新前沿和开放高地。深入实施创新驱动发展战略，推进科技创新十大工程，培育一批具有核心竞争力和创新能力的领军型企业，打造一批创新创业综合体，引进一批研发创新团队。加强与著名大学、科研院所合作，共建研发中心、孵化基地、人才培养基地等；依托本地院校，大力发展职业教育，进一步浓厚“大众创业、万众创新”氛围。加强对外开放平台和口岸建设，创建自贸区异地共建区，强化通关便利性建设，依托保税物流中心建设商丘综合保税区，依托铁路物流基地建设铁路一类口岸，全力打造开放型经济和枢纽经济发展的新载体，形成引领经济发展的新引擎。

　　－－基本建成民生保障能力显著提升的幸福城市。实现更加充分的就业，城乡居民收入有较大幅度增长，10分钟便民生活圈基本形成，社区精神文化生活更加丰富，医养健康业得到充分发展。教育医疗、体育休闲、文化娱乐等公共产品供给能力显著增强，社会保障能力和水平不断提升，社会更加和谐稳定，不断增强人民群众的获得感、安全感、幸福感，不断提升人民群众的认同感、归属感、自豪感。

　　各位代表！商丘发展的接力棒传到了我们手里，这是人民群众的殷切期盼，是历史赋予的光荣使命，更是我们义不容辞的责任担当！站在新的历史起点上，把未来五年的宏伟蓝图变为现实，离不开全市900多万人民的齐心协力、团结实干。我们相信，经过五年的不懈奋斗，一个更有实力、更具活力、更富魅力、更加幸福的商丘必将展现在全市人民面前！

　　 三、2017年重点工作

　　今年是党的十九大胜利召开之年，是国家实施供给侧结构性改革的深化之年。做好今年的工作，对于巩固“十三五”良好开局，夯实全面建成小康社会基础，实现又好又快跨越发展至关重要。

　　今年全市经济社会发展主要预期目标是：生产总值增长8．5％；一般公共预算收入增长8．5％；全社会固定资产投资增长15％；社会消费品零售总额增长12％；实际利用省外资金增长7％左右；城乡居民收入高于经济增长速度；居民消费价格涨幅3％左右；节能减排等约束性指标完成省定目标。实现上述目标，我们将按照又好又快跨越发展的总要求，以九项跨越发展行动计划和2017年行动方案为抓手，重点实施339个重大项目，完成年度投资1630亿元。主要抓好十个方面的工作：

　　（一）以产业集聚区二次创业为契机，加快工业经济转型发展。以壮大产业规模、完善产业链条、提升产品科技含量和附加值为关键，以引进和实施重大项目为载体，以晋位升级为目标，强力推进产业集聚区转型发展。全市产业集聚区完成固定资产投资1300亿元以上，规模以上工业企业实现主营业务收入2600亿元以上。力争6月底前与恒天集团等重点纺织印染企业签定入园合同，加快永安纺织等重点企业改扩建工程建设，带动纺织服装业加快发展，早日实现20万吨染整产能目标。深化与海航、万宝等企业集团合作，推进冷藏集装箱等项目尽快开工建设，推动冷藏运输制造业加快向系列化、规模化、智能化转变。确保一期投资10亿元的华晶金刚石智能化生产项目年内建成投产，在壮大生产规模的同时，深化金刚石应用领域的研发创新合作，整合产业物流配送资源，拓展市场营销渠道，提升产品竞争力和企业效益。确保环保电镀中心项目年底前分期运营。加快特步、赛琪二期等17个纺织服装制鞋项目，上海梅林肉食加工等19个食品业项目，中天药业、哈森二期等5个医药项目，富锦电子、金振源二期等9个电子信息项目建设进度，确保如期开工或竣工达产，高新技术企业和先进制造业占比有较大提升。4个省级高新区突出发展以生物医药、制冷装备、工量具制造、新材料、新能源为主的高新技术产业，争创国家级高新区；开发区加快10平方公里先进装备制造业园区起步区建设步伐，争取浩天集团8月底前，西奥智能电梯、神火铝箔等项目11月底前开工建设，年内力争汤姆逊等20家电子信息产业项目入驻电子信息产业园，加快先进装备制造、电子信息、现代物流等产业发展，争创国家级经开区。继续推动钢铁、煤炭行业化解过剩产能，彻底关停拆除5个“地条钢”生产企业，超额完成105万吨煤炭化解任务。加快产业集聚区道路、电力、通信、供热、污水处理等基础设施建设，10月底前，具有一定用热需求规模的产业集聚区实现集中供热，完成“双替代”目标。推动综合服务、检验检测、信息咨询、技术研发等公共服务平台建设，完善教育、医疗、文化、娱乐和商业等配套设施，提升综合承载能力，推动产城融合发展。搞好企业服务，切实解决企业发展中的融资、用地、用工等难题，筹办好食品、针织服装和制冷博览会，帮助企业开拓市场。

　　（二）以“六六三三”晋位争优为目标，坚决打好脱贫攻坚战。把脱贫攻坚放在统揽经济社会发展全局的战略地位，科学谋划，统筹推进，狠抓落实，确保实现全市“6633”晋位争优目标。力争统筹整合各类资金20亿元，加快实施产业扶贫项目，落实“一乡一园、一村一点”要求，做到扶贫车间贫困村全覆盖。着力抓好金融扶贫，扶贫再贷款余额达到10亿元以上，扶持增收政策覆盖64．6万贫困人口，实现转移就业2．5万人。落实健康扶贫各项措施，力争所有贫困村都配备一个标准化卫生室和一名全科医生，减少群众因病致贫、因病返贫。巩固教育扶贫成果，确保贫困家庭学有所教。做好贫困村基础设施建设，对全市842个贫困村，继续实施“九大基础工程”和“六改工程”，从根本上改变贫困村和贫困家庭的落后面貌。通过以上精准扶贫措施，确保7．38万农村贫困人口稳定脱贫，决不让一个贫困人口在全面建成小康社会的道路上掉队。

　　（三）以种养业结构调整为突破，提升农业产业化发展水平。以土地流转促结构调整，以规模化种植提高农业机械化水平，实施农业“八大增收工程”，增强抵御自然灾害和市场风险能力。持续推进高标准粮田建设，新建高标准粮田60万亩，确保粮食产量稳定在120亿斤以上；适当调减玉米种植面积，大力发展优质小麦、优质花生、优质草畜和优质林果，扩大食用菌、三樱椒、酥梨、无公害蔬菜等种植面积，加强区域特色农产品品牌建设。促进农产品加工业发展，增加优质农产品加工品种供给，新认定市级农业产业化龙头企业10家以上。加快牛、羊、生猪等畜牧标准化养殖小区建设，各县（区）重点培育发展1～2个现代畜牧产业化集群，加快推进正大集团、正邦集团生猪全产业链项目和科迪乳业、花花牛乳业、宏星牧业养殖基地建设。促进农业与现代服务业融合发展，研究出台促进土地流转的政策措施，推动土地集中规模经营，新发展专业合作社500家、家庭农场300个、种粮大户200家。培育3～5家本土农业电商企业，市级以上农业产业化龙头企业电子商务率达到60％，打造10个特色农产品电子商务示范乡镇、20个示范村（基地）。加强重点水利工程建设，实施病险水闸除险加固、中小河流治理和三义寨引黄灌区续建配套项目，新增改善节水灌溉和除涝面积80万亩。

　　（四）以电商和物流业为先导，带动枢纽经济加快发展。把电子商务尤其是跨境电子商务和现代物流业，作为发展枢纽经济的先导产业，力争保税物流中心8月底前封关运营，年底前跨境交易额完成10亿元以上。加快完成铁路物流基地选址和土地预审等工作，6月底前完成铁路一类口岸的编制、申报等基础性工作，争取尽快立项建设。深化与海航集团在冷链物流等方面的合作，启动临空经济区规划研究。出台促进电子商务加快发展的扶持政策，每个县（区）规划建设1个电子商务园区，争创2个省级跨境电商示范园区，新增跨境电商应用企业50家。深化与阿里巴巴集团合作，新增线上企业200家，分行业建设跨境电商网上基地。瞄准物流龙头企业开展招商，力争签约10亿元以上的物流项目1个、5亿元以上的2个、3亿元以上的5个；加快传化物流、中部电商产业园、中原佳海、双汇冷链物流等9个重点项目建设，力争传化物流一期11月投入运营；力争新加坡丰树物流园、香港胜记仓豫东商谷、华东商储产业园3个项目签约落地。举办好高层次物流和枢纽经济发展论坛。

　　（五）以“三大主战场”为载体，加快现代服务业发展。加快古城历史文化旅游创意创新区建设。加强古城业态研究，编制好创意创新区规划，依照古城保护条例，做好各项工程的前期准备工作，在保护的基础上进行修复与展示。在挖掘古城特有历史文化上下功夫，形成以燧皇取火、玄鸟生商、阏伯观星为主的历史故事系列，以古城池建设、家风家教文化为主的博物馆展示系列，以应天书院、文庙为主的国学教育系列，以闹龙街、侯方域和李香君爱情故事为主的名人轶事系列等，形成魅力独具的展示体系。选择有实力、有品牌、有市场、有国际影响力的企业进行策划经营，着力打响“游商丘古都城、读华夏文明史”的旅游品牌。加快商务中心区建设。以日月湖周边6平方公里为核心，加快文化艺术中心等9个在建项目工程进度，确保绿地星级酒店、豫东设计创意中心、建业商务办公等8个项目年内开工建设。加快高铁商贸区建设。以4．5平方公里核心区为重点，尽快开工建设高铁站北广场至人民公园南大门绿轴及步行街工程，延伸城市中轴线，开工建设红星美凯龙星艺佳、爱琴海和万洋国际等城市综合体，带动道北区域加快发展。积极发展金融业，争取新引进6家金融机构；力争1家企业在海外主板上市，2家以上企业在新三板挂牌，60多家企业在中原股权交易中心挂牌；规范发展各类融资性担保公司和小贷公司，构建多元化融资模式，解决中小企业资金难题。

　　（六）以综合交通枢纽建设为关键，增强城镇基础承载能力。加快商丘铁路综合枢纽规划编制，进一步明确商丘站、商丘南站、新区站的功能定位；确保商合杭高铁顺利建设；积极配合铁路部门，完成京九高铁霸商段各项前期工作，力争明年上半年开工建设；确保新区站站房及站前广场如期开工建设，力争启动商丘站南站房改造工程，积极协调配合铁路部门尽快完成京九高铁商丘动车所选址，加快推进高铁换乘中心工程，10月底前投入使用。力争开工建设商丘机场；完成沱浍河航运桥梁改造，争取开工航道工程。“三环九放射”中的中心城区至夏邑永城、宁陵睢县、柘城、曹县等5条道路力争年内开工建设，启动北海路与商周高速互通立交工程建设；10月底前完成庄周大道、清凉大道等内环物流通道一期工程；按照“窄马路、密路网”的原则，完善中心城区路网体系，加快建设北海路西延、神火大道南延等主城区十纵十横主干道工程，加快189条城市支路网、314个公交港湾、10个停车场建设，畅通微循环，缓解停车难。在加快水源地建设的同时，8月底前开工建设第四水厂扩建工程，新增供水能力20万吨，改进提升现有水厂处理工艺，完善监测机制，改善供水质量；确保第八污水处理厂6月底前投运，污水处理能力达到43万吨，同步铺设污水管网188公里，启动第二污水处理厂建设，提升污水收集处理能力；10月底前完成丰源热电超洁净排放和新增机组建设任务，新增供热能力500万平方米，同步完成热网建设176公里；完成燃气管网136公里建设任务。巩固“六城”联创成果，围绕创建全国卫生城市和文明城市，不断提升城市建设管理水平。按照省定目标，在坚持“四不拆”的前提下，解决好拆迁、过渡周转、企业搬迁等问题，为道路、公园、学校、医院等建设和水系治理创造条件，26个安置点、3．8万套安置房6月底之前全部开工建设。坚持“房子是用来住的，不是用来炒的”，加强商品住宅用地供应，统筹好住房供给体系，稳定市场预期，促进房地产行业平稳健康发展。

　　扎实推进百城建设提质工程。认真落实省委、省政府提出的重大战略举措，把“百城建设提质工程”作为加快城镇化进程、吸纳产业和人口转移就业的重要载体，按照以人为本、生态优先、基础先行、功能复合、文化突出、特色彰显的要求，贯彻落实好新发展理念。加快永城区域副中心城市建设步伐，不断增强吸纳产业与人口转移能力；夏邑按照试点县的标准，率先发展。各县9月底前全部完成县域城乡总体规划编制，同步编制专项规划和城市设计，“六线”控规实现全覆盖。统筹推进县城供水、热力、燃气、污水、垃圾处理、水系公园等市政设施和医疗、教育、文化等公共服务设施建设，提升县城综合承载能力。出台鼓励人口转移的户籍政策、社会保障政策等，新增农业转移13．5万人以上；建立完善融资和政府债务风险管控机制，创新融资模式，吸引社会资本参与百城建设提质工程；加强县城管理，不断完善精细化管理措施，提高群众的满意度；展现城镇特色，将历史文化元素融进城镇规划建设，塑造城镇风貌，彰显城镇个性。着力抓好28个重点镇建设，打造一批各具风情的特色小镇。有序推动新农村建设，分类妥善解决新型农村社区遗留问题，年内完成60％的整改任务。继续开展“美丽商丘·整洁村镇”活动，加强农村垃圾、污水处理设施建设，所有行政村建立清扫保洁和垃圾分捡机制。

　　（七）以环境污染防治攻坚为抓手，改善城乡生态环境。环境污染防治既是全省重要攻坚战，也是优化发展环境、提升发展质量的现实选择。我们将牢固树立“绿水青山就是金山银山”、生态环境就是发展环境的理念，持续打好环境污染防治攻坚战。巩固提高道路扬尘治理成果，落实拆迁管理和渣土运输“两固定、四严格”措施，全面实现重点企业在线监控，坚决遏制“黑加油站点”反弹，做好秸秆禁烧和综合利用工作，保持大气质量持续好转的态势，确保完成省定目标任务，让“商丘蓝”成为常态。加强水环境治理，中心城区九条河6月底前完成河道两侧污水管网建设和截污纳管工程，8月底前完成河岸护坡和两岸部分景观工程，年底前完成中水循环利用管网铺设工程，全部完成禁养区养殖企业关停搬迁，按期完成沱河、惠济河、东沙河、包河、大沙河等治理，确保流域和过境水质断面长期达标。以黄河故道、区域河流两岸为主体，完成11．7万亩绿化任务。中心城区开工建设“一园、两桥、三廊、六河、12个便民公园、14块绿地、68条道路”等园林绿化项目。加强水源建设，加快引黄移动泵站和闫谭提水工程建设，实施林七、吴屯水库清淤扩容，配合做好引江济淮前期工作，促进水资源可持续利用。积极开展土壤污染防治，开展土壤环境质量调查，实施农用地分类管理，保障农业生产环境安全。

　　（八）以改革创新为引领，增添发展新动力。以智慧城市建设为载体，深入推进行政管理体制改革，再取消、下放一批行政审批事项，清理规范中介服务，6月底前基本建成市县乡村四级网上审批服务体系，实现“一号申请、一窗受理、一网通办”，最大限度方便企业和群众办事。全面落实政府部门权责清单和负面清单制度，实现“两随机一公开”监管方式全覆盖。加快商事制度改革，推行“多证合一、一照一码”、全程电子化，建立支持实体经济发展的快速通道。持续打好国企改革攻坚战，有序推进神火集团、路桥集团、市政工程总公司等企业改革，完善法人治理结构，规范经营管理，增强企业发展活力。深化财税体制改革，认真落实营改增收入划分方案，提高财政基本保障能力；完善政府性债务风险防控措施，确保不发生政府性债务风险；深化投融资体制改革，发挥财政资金的撬动作用，广泛应用政府与社会资本方合作模式（PPP），拓宽融资渠道。全面完成农商行组建工作。统筹推进医疗、医保、医药联动改革，全面推进县级公立医院综合改革，有序实施分级诊疗制度，取消药品价格加成，推行按病种付费为主、多种付费方式相结合的医保支付方式；深化计生服务管理改革，促进人口均衡发展。完成城市管理综合执法改革任务，加快盐业、供销、国土执法、国有林（农）场等改革步伐。全面完成土地承包经营权和农村宅基地及房屋确权登记颁证，落实承包土地所有权、承包权、经营权分置政策。加大承接产业转移力度，强力实施开放招商，瞄准长三角、珠三角、京津冀等区域，精准定向招商，提升招商实效，提高合同履约率、资金到位率、项目开工率。力争全年落地3亿元以上项目50个以上，每个县（区）达到5个以上；全市利用省外资金突破600亿元，继续保持全省先进位次。深入推进创新驱动发展战略，新增高新技术企业10家、省级科技型中小企业30家以上。设立扶持资金，重点支持优势企业建立工程技术研究中心、重点实验室、院士工作站等研发机构，年内申报省级工程技术研究中心8家、省级科技企业孵化器1家。每个产业集聚区至少建设1个创新创业综合体，4个高新区都要建成1个国家级科技企业孵化器。探索建立商丘科学院，助推产业转型发展。

　　（九）以提升公共服务水平为目标，推进社会事业全面发展。着力补齐短板、提升水平，优化基本公共服务供给，不断夯实共享发展基础。把区域教育中心建设作为阶段性重点工作，实施优质教育资源倍增计划，力争医专、市一中、市实验幼儿园新校区今年秋季招生。加快安置区学校建设，支持规范民办教育，大力发展职业教育。完成“全面改薄”任务，改扩建中小学555所、幼儿园91所、农村寄宿制学校93所；落实“两免一补”政策，实现教育经费随学生流动，决不让贫困阻挡孩子求学上进的脚步。规范实施国家基本公共卫生服务项目，扩大受益人群覆盖面，完成209个村室、23个乡镇卫生院建设任务，促进基本公共卫生服务均等化。扩大健康签约服务试点范围，加快6个县医联体建设，提升远程诊疗水平。推进重点医疗设施建设，市第一人民医院儿童病房楼10月底前投入使用，依托商丘医专老校区，建设市第一人民医院心脑血管、肿瘤和康复分院；实施市第三人民医院搬迁，市第四人民医院新院区投入使用，市妇幼保健院年底前主体完工，示范区利君医院和市中医院新院区分别于7月底、10月底前开工建设，不断满足群众就医需求。落实养老服务政策，发展健康养老产业，支持宁陵等县建设健康医养社区。结合公共绿地、社区服务中心建设，广布健身器材，扩大全民健身运动成效。大力实施文化惠民、公共文化设施提升等工程，做好市规划馆、图书馆、方志馆和市民中心、市青少年活动中心前期工作，力争年内开工建设。加强村庄和社区文化广场建设，继续实施“舞台艺术送农民”“百戏进百乡”和“农村电影放映工程”。整合域内文化旅游资源，把商文化、火文化、汉梁文化、根亲文化等发掘好、展示好、传承好，实施黄河故道生态旅游规划建设，做好大运河文化遗产保护与展示，优化旅游线路，推进以历史文化为核心的全域旅游，立足商丘，对接周边，站位全国，着力打造“一带一路”黄金旅游线上的新亮点。

　　（十）以保障改善民生为根本，确保社会和谐稳定。不断加大投入，办好民生实事，增强人民群众获得感。深入实施全民技能振兴工程，努力扩大就业，着力做好高校毕业生、农村劳动力、“零就业家庭”等重点群体就业工作，全年城镇新增就业5．5万人。完善社会保障体系，进一步扩大各类社会保障覆盖面，加强农村“三留守”人员关爱和困境儿童保障，城乡居民基本养老保险基础养老金最低标准提高到每人每月80元，城乡居民基本医保财政补助标准提高到平均每人每年450元。深化机关和事业单位养老保险制度改革，落实好退休人员基本养老金政策。开工建设4个县级残疾人基础服务设施，中心城区实行残疾人免费乘坐公交车。加快推进气象现代化建设，全面提升防灾减灾能力。加强社会治理创新，做好法律援助工作，开展重大项目社会稳定风险评估，预防化解各类社会矛盾。强化社会治安综合治理，完善反恐和打击犯罪机制，确保社会和谐稳定、人民安居乐业。普及校园安全知识，防范校园暴力。完成食品药品检验检测中心建设，推进食品安全市县创建，确保群众“舌尖上的安全”。严格落实安全生产责任制，强化对建筑施工、道路交通、消防等重点领域安全治理，坚决遏制重特大事故发生。

　　加强社会主义核心价值观教育，深化行业道德建设和群众性精神文明创建，继续讲好“商丘故事”，壮大“商丘好人”群体，启动“商丘好人”主题公园建设，提升文明素质，凝聚推动发展的强大精神力量。深入开展“七五”普法教育，形成全社会尊法学法守法用法的氛围。加强国防教育和国防后备力量建设，支持国防和军队改革，支持驻商解放军、武警部队、预备役部队、民兵和人民防空建设，做好军队转业干部、退役士兵的接收安置，继续落实好随军家属就业就学政策，推进军民深度融合发展，争创省双拥模范城“八连冠”。促进民族团结、宗教和睦。支持工会、共青团、妇联等人民团体发挥作用。全面做好统计、外事侨务、对台、社科研究、档案管理、农林科研、机关事务管理和援疆等工作。

　　 四、努力建设人民满意的政府

　　各位代表！新起点、新蓝图为发展指明了前进方向，新形势、新任务对工作提出了更高要求。全市政府系统必须牢固树立“四个意识”，牢记人民政府为人民的宗旨，严守政治纪律政治规矩，立党为公、执政为民，努力建设人民满意的政府。

　　（一）着力真抓实干，建设务实政府。把务实作为政府工作的基本要求，一切工作从商丘实际出发，坚决不要急功近利的短期行为，坚决不做虚报浮夸的表面文章，坚决不搞劳民伤财的形象工程。把实干做为政府工作的基本规范，大力精简会议和文件，坚决杜绝以会议落实会议、以文件落实文件的不良风气。对确定的工作任务，勇于担当，主动作为，一件一件抓落实，一项一项求突破，确保把各项决策部署落到实处，以抓落实的实际效果展现政府工作的公信力和执行力。

　　（二）着力依法行政，建设法治政府。坚持把法治精神、法治思维、法治方式融入政府工作全过程，自觉接受人大法律监督、政协民主监督和社会监督，认真办理人大代表建议和政协委员提案。坚持依法科学民主决策，落实重大行政决策法定程序，确保行政行为合法依规。加强服务型执法建设，落实行政执法责任制，提升服务型执法水平。科学编制政府立法计划，推动城乡建设管理、水资源保护、中小学规划等重点领域立法。规范行政执法行为，严格公正文明执法。

　　（三）着力转变职能，建设服务政府。不断深化行政管理体制改革，突出制度创新，完善运行机制，推动政府职能向创造良好发展环境、提供优质服务、维护社会公平正义、提高社会管理水平转变，减少政府的自由裁量权，增强市场的自主选择权。强化政府公共服务职能，努力补齐文化、教育、医疗、社会保障等发展短板，为群众提供更多的公共产品和公共服务。大力推行政务公开和政府信息公开，依法保障群众的知情权、参与权、监督权，让人民监督权力，让权力在阳光下运行。

　　（四）着力改进作风，建设高效政府。全面加强政府系统作风建设，认真开展懒政怠政、为官不为专项监督工作。从市政府领导班子做起，切实促进“马上办抓落实”常态化。密切联系群众，深入基层开展调查研究，着力解决实际问题。完善督促检查机制，严格绩效考核，健全督查问责机制，打通政策落实的梗阻与障碍。推动政府管理创新、制度创新、工作创新，确保政府工作更富创造性、科学性。扎实推进“两学一做”学习教育常态化、制度化，努力建设一支高素质的公务员队伍，不断提振干事创业的精气神、汇聚团结奋进的正能量。

　　（五）着力从严治政，建设廉洁政府。严格遵守党的廉洁自律准则和各项纪律要求，以更高的标准、更严的举措正风肃纪，促进政府清廉、干部清正、政治清明。强化对政府投资、公共资源交易等重点领域的监管，坚决惩治侵害群众利益的不正之风和腐败问题。自觉践行“三严三实”要求，坚决落实中央八项规定精神，驰而不息纠正“四风”，厉行勤俭节约，反对铺张浪费，严控“三公”经费。强化行政监察和审计监督。坚持用制度管权、管事、管人，始终做到忠诚、干净、担当，从源头上预防腐败。

　　各位代表！时代开启新征程，实干谱写新篇章。让我们更加紧密团结在以习近平同志为核心的党中央周围，在省委、省政府和市委的坚强领导下，撸起袖子加油干，扑下身子抓落实，凝心聚力，砥砺前行，为实现商丘经济社会又好又快跨越发展、决胜全面小康做出新贡献，以优异成绩迎接党的十九大胜利召开！
